# Cyclopædia of the Diseases of Children, Medical and Surgical

**Published:** 1889-06

**Authors:** 


### Book Reviews.

---

CYCLOPÆDIA OF THE DISEASES OF CHILDREN, MEDICAL AND SURGICAL. By JOHN M. KEATING, M. D. Vol. I. Philadelphia: J. B. Lippincott Co., 1889.

We have received the above named work. In it the editor has endeavored to place before students and practitioners a ready reference on the care and treatment of children. Added to the recent researches in bacteriology, the minute details of diagnosis, the great attention paid to the essentials of practical medicine and management of children make it a complete and comprehensive volume, and we take great pleasure in commending the work to the medical profession as eminently worthy of their consideration.

